# Cell-Free Culture Supernatant of *Bifidobacterium breve* CNCM I-4035 Decreases Pro-Inflammatory Cytokines in Human Dendritic Cells Challenged with *Salmonella typhi* through TLR Activation

**DOI:** 10.1371/journal.pone.0059370

**Published:** 2013-03-12

**Authors:** Miriam Bermudez-Brito, Sergio Muñoz-Quezada, Carolina Gomez-Llorente, Esther Matencio, Maria J. Bernal, Fernando Romero, Angel Gil

**Affiliations:** 1 Department of Biochemistry and Molecular Biology II, Institute of Nutrition and Food Technology “José Mataix”, Biomedical Research Center, University of Granada, Granada, Spain; 2 Global Centre for Child Nutrition Technology, Hero Group, Alcantarilla, Murcia, Spain; Istituto Superiore di Sanità, Italy

## Abstract

Dendritic cells (DCs) constitute the first point of contact between gut commensals and our immune system. Despite growing evidence of the immunomodulatory effects of probiotics, the interactions between the cells of the intestinal immune system and bacteria remain largely unknown. Indeed,, the aim of this work was to determine whether the probiotic *Bifidobacterium breve* CNCM I-4035 and its cell-free culture supernatant (CFS) have immunomodulatory effects in human intestinal-like dendritic cells (DCs) and how they respond to the pathogenic bacterium *Salmonella enterica* serovar *Typhi*, and also to elucidate the molecular mechanisms involved in these interactions. Human DCs were directly challenged with *B. breve*/CFS, *S. typhi* or a combination of these stimuli for 4 h. The expression pattern of genes involved in Toll-like receptor (TLR) signaling pathway and cytokine secretion was analyzed. CFS decreased pro-inflammatory cytokines and chemokines in human intestinal DCs challenged with *S. typhi*. In contrast, the *B. breve* CNCM I-4035 probiotic strain was a potent inducer of the pro-inflammatory cytokines and chemokines tested, i.e., TNF-α, IL-8 and RANTES, as well as anti-inflammatory cytokines including IL-10. CFS restored TGF-β levels in the presence of *Salmonella*. Live *B.breve* and its supernatant enhanced innate immune responses by the activation of TLR signaling pathway. These treatments upregulated *TLR9* gene transcription. In addition, CFS was a more potent inducer of *TLR9* expression than the probiotic bacteria in the presence of *S. typhi*. Expression levels of *CASP8* and *IRAK4* were also increased by CFS, and both treatments induced *TOLLIP* gene expression. Our results indicate that the probiotic strain *B. breve* CNCM I-4035 affects the intestinal immune response, whereas its supernatant exerts anti-inflammatory effects mediated by DCs. This supernatant may protect immune system from highly infectious agents such as *Salmonella typhi* and can down-regulate pro-inflammatory pathways.

## Introduction

Probiotic bacteria including lactobacilli and bifidobacteria are part of a normal intestinal microbiota in humans and generally considered as potentially beneficial to various aspects of host metabolism [1]. *Bifidobacterium* sp. are among the most relevant probiotic microorganisms because they colonize the intestinal tract soon after birth, are present at high levels in the guts of infants and adults and promote beneficial effects on intestinal ecology and immune responses [2], [3]. Several mechanisms for the favorable influence of probiotic bacteria on the intestinal mucosa have been suggested including the secretion of antimicrobial products, resistance to pathogen colonization, barrier function enhancement and maintenance, modulation of epithelial cell signal transduction and innate and adaptive immunomodulation [3], [4]. The beneficial effects of specific probiotic strains have been established for the treatment and prevention of many diseases [5], including diarrhea [6], the alleviation of lactose intolerance [7] and postoperative complications [8], antimicrobial [9] and anticolorectal cancer activity [10], [11] and for reducing irritable bowel symptoms [12] and increasing the relapse time for some inflammatory bowel diseases [13].

The probiotic properties of commensal bacteria including lactobacilli and bífidobacteria are likely to be determined at least in part by their effects on dendritic cells (DCs) [1], a complex, heterogeneous group of multifunctional antigen-presenting cells (APCs) that comprise a critical arm of the immune system [14], [15]. These cells play a critical role in the orchestration of the adaptive immune response by inducing tolerance and adaptive immunity. Understanding the direct interaction between commensal bacteria and DCs is particularly important to know how the immune system of the gut is locally able to distinguish these bacteria from pathogens and to elicit a tolerogenic response [16]. The primary response to these bacteria is triggered by the innate pattern recognition receptors (PRRs), which bind pathogen-associated molecular patterns (PAMPs). PRRs comprise Toll-like receptors (TLRs), nucleotide-binding oligomerization domain (NOD)-like receptors (NLRs), adhesion molecules and lectins [4], [17]. The binding of microbe-associated molecules to these receptors can activate APCs and initiate a signaling transduction cascade that leads to the release of cytokines and initiation of the acquired immune response [18].

Mucosal DCs appear to have unique properties that distinguish them from peripheral DCs [19]. However, to date, probiotic activity has often been tested in monocyte-derived DCs (MoDCs) or murine DCs, which are quite different from human gut DCs [20]. For this reason, in this study, we used intestinal-like human DCs that were developed from umbilical cord blood CD34+ progenitor cells. These human DCs are Langerhans-like cells that extend dendritic processes and sample antigens similarly to the lamina propria DCs in the gut that sample luminal antigens [21].

We have previously reported some of the probiotic properties of *Bifidobacterium breve* CNCM I-4035, a novel bifidobacteria strain isolated from the feces of newborns that were exclusively breast-fed [22], [23]. In the present work, we studied the immunomodulatory effects of *B.breve* CNCM I-4035 and its cell-free culture supernatant (CFS) on human intestinal-like DCs and how the treated DCs interact and respond to the pathogenic bacteria *Salmonella enterica* serovar *Typhi* at molecular level.

## Materials and Methods

### Ethic Statement

The ethical Committee of Granadás University approved this study.

*Bifidobacterium breve* was obtained from feces of breast-fed newborns, in a previous work [24]. Briefly, 12 healthy, exclusively breast-fed infants, aged 1 month, were selected for the study at the Clinic Hospital of the University of Granada. This study was conducted according to the guidelines laid down in the Declaration of Helsinki and all procedures involving human subjects were approved by the Ethical Committee of the University of Granada. Written informed consent was obtained from the parents after a careful explanation of the nature of the study.

### Preparation of bacteria and cell-free culture supernatant

*B. breve* CNCM I-4035was isolated from the feces of breast-fed newborns and previously selected for its *in vitro* probiotic characteristics [22], [23]. *B. breve* CNCM I-4035 was routinely anaerobically cultured for 24 hours at 37°C in de Man-Rogosa-Sharpe (MRS) broth medium (Oxoid, Basingstoke, United Kingdom) supplemented with 0.05% (wt/vol) cysteine (Sigma-Aldrich, St. Louis, MO) to promote the growth of *B. breve*. The supernatant of the culture medium was collected by centrifugation at 12,000×*g* for 10 min, neutralized to pH 7.0 by the addition of 1 N NaOH and concentrated ten-fold by lyophilization. The supernatants were passed through a 0.22-μm pore size filter unit (Minisart hydrophilic syringe filter; Sartorius Stedim Biotech GmbH, Goettingen, Germany) and stored at −20°C until use. The supernatant was added to the DC culture medium at a concentration of 7% v/v.

*Salmonella enterica* serovar *Typhi* CECT 725 was provided by the Spanish Type Culture Collection (CECT; Burjassot, Spain) and aerobically cultured in tryptone soy broth (Panreac Química, Barcelona, Spain).

For experiments, *S. typhi* was cultured for 8 h at 37°C in tryptone soy broth and then subcultured 1:500 in RPMI 1640 medium (Sigma-Aldrich) containing 10% fetal bovine serum (FBS; Gibco Invitrogen, Paisley, United Kingdom) at 37°C overnight.

### Cell preparation

DCs generated from umbilical cord blood CD34^+^ progenitor cells (hematopoietic stem cells) were supplied by MatTek Corporation (Ashland, MA). These cells were seeded in 24-well plates in DC maintenance medium (DC-MM; MatTek) containing cytokines and antibiotics.

### Bacterial co-culture and DC stimulation

Cell cultures were seeded in 24-well plates at a density of 2×10^5^ DCs/well. For incubations, DC-MM was replaced with RPMI-1640 medium. DCs were co-incubated with *B. breve* CNCM I-4035 bacteria (10^7^ CFU/ml) or CFS as well as *S. typhi* (10^6^ CFU/ml) or a combination of these treatments for 4 h at 37°C in a 5% CO_2_/95% air atmosphere. After incubation, the DCs were washed with PBS, and DC-MM (containing cytokines and antibiotics) was added to the wells and incubated for an additional 20 h. Cell supernatants and cells were collected for cytokine analysis and RNA extraction, respectively. *Escherichia coli* lipopolysaccharide (LPS; Sigma-Aldrich) was applied at a concentration of 20 ng/ml as a positive control. Negative-control cultures contained unstimulated DCs.

### Cytokine and chemokine quantification in culture supernatants

Cytokine production was measured by immunoassay with the MILLIplex^TM^ kit (Linco Research Inc., MO) using the Luminex 200 system according to the manufacturer's instructions. IL-1β, IL-6, IL-8, IL-10, IL-12(p40), IL-12(p70), TNF-α, IFN-γ, MCP-1/CCL2, MIP-1α/CCL3, RANTES/CCL5, MDC/CCL22 and TGF-β were analyzed. We performed three independent experiments.

### Reverse transcriptase (RT) reaction and polymerase chain reaction (PCR)

Total RNA was isolated from cells using the RNAqueous Kit (Ambion, Paisley, United Kingdom) and additional Turbo DNase treatment (Ambion) according to the manufacturer's recommendations. The RNA quality was verified using a Model 2100 Bioanalyzer (Agilent, Santa Clara, USA), and the RNA concentration was determined using a Rediplate 96 Ribogreen RNA Quantitation Kit (Gibco, Invitrogen). The total RNA was reversed-transcribed using an RT^2^ First-strand Kit (SABiosciences Corporation, Frederick, MD). Real-time PCR was performed using an RT^2^ Real-time PCR SYBR Green/ROX Kit (SABiosciences) on an ABI Prism 7500 sequence detector (Applied Biosystems, Foster City, CA). Real-time RT-PCR analysis of the samples was performed using a Human TLR Signaling Pathway PCR Array (SABiosciences), which includes primer pairs specific for the following 20 genes related to TLR-mediated signaling pathways: *TLR1, TLR2, TLR3, TLR4, TLR5, TLR9, MYD88, TNF-α, IRAK-1, IRAK-4, TOLLIP, CASP8, IL-10, TAK-1, JNK, NFKBIA, NFKB-1, TBK-1, MAPK14* and *IRF-3*. The housekeeping gene *GAPDH* was used as a control. The thermal profile for all reactions was: 1 cycle of 95°C for 10 min and 40 cycles of 95°C for 15 s and 60°C for 1 min. The expression levels of the target genes were normalized to those of untreated DCs (control).

### Statistical analysis

The results shown are the mean ± SEM of three independent experiments.

The differences in cytokine levels and gene expression between treatments were compared using the Mann-Whitney *U*-test. Analyses were performed using NCSS 2007 software (Kaysville, UT). P<0.05 was considered significant and is indicated with an asterisk in the figures.

Differences between DCs treated with *Salmonella* and *Salmonella* plus *B. breve* CNCM I-4035 or its CFS were also evaluated. P<0.05 was considered to significant and is indicated in the figures with a pound sign (#).

## Results

### Supernatant of *B. breve* CNCM I-4035 decreases cytokine release in human DCs co-cultured with *S. typhi*

**Figure 1:**
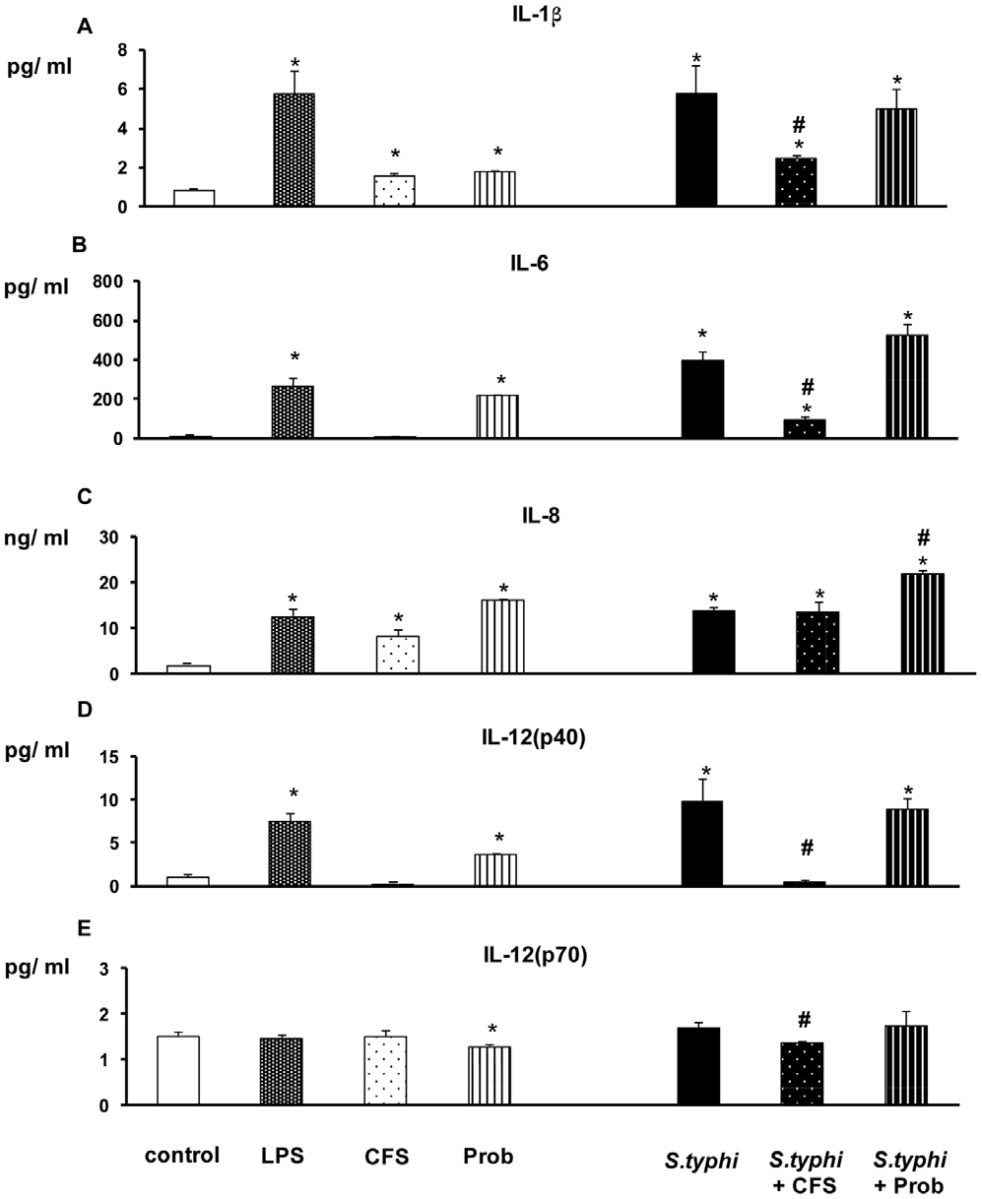
Effects of *B. breve* CNCM I-4035 and its cell-free culture supernatant on the secretion of pro-inflammatory cytokines by intestinal-like human dendritic cells. Dendritic cells (DCs) were incubated for 4 h with the *B. breve* CNCM I-4035 probiotic (Prob) or its cell-free supernatant (CFS), *Salmonella* (Sal) or both and further incubated for 20 h in medium containing antibiotics. *E. coli* lipopolysaccharide (LPS; 20 ng/ml) was used as a positive control. Negative-control cultures contained unstimulated DCs. Culture supernatants were collected, and the cytokine levels were assessed using an immunoassay. The production of IL-1β, IL-6, IL-8, IL-12(p40) and IL-12(p70) was measured. The data shown are the mean value ± SEM for three independent experiments. *, p<0.05 compared with the negative control; #, p<0.05 compared with *S. typhi*; N.D. indicates not detected.

**Figure 2:**
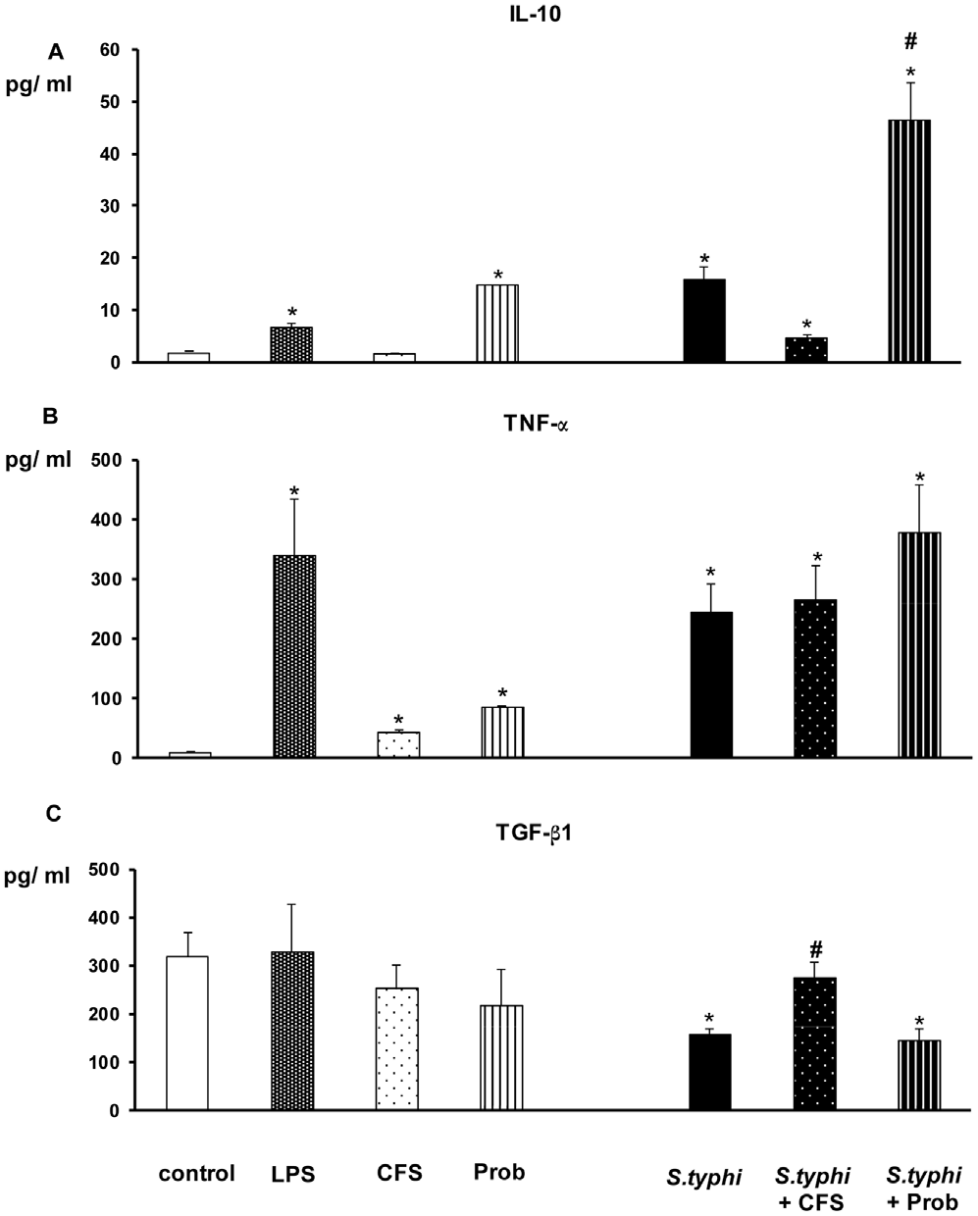
Measurement of anti-inflammatory cytokines and TNF-α in DCs after exposure to *B. breve*, *Salmonella* or a combination of the two. Dendritic cells (DCs) were incubated for 4 h with the *B. breve* CNCM I-4035 (Prob) probiotic or its cell-free supernatant (CFS), *Salmonella* (Sal) or both and then incubated for 20 h in medium containing antibiotics. *E. coli* lipopolysaccharide (LPS; 20 ng/ml) was used as a positive control. Negative-control cultures contained unstimulated DCs. Culture supernatants were collected, and the cytokine levels were assessed by an immunoassay in which the production of IL-10, TNF-α, TGF-β1 and TGF-β2 was measured. The data shown are the mean value ± SEM of three independent experiments. *, p<0.05 compared with controls; #, p<0.05 compared with *S. typhi*; N.D. indicates not detected.

**Figure 3:**
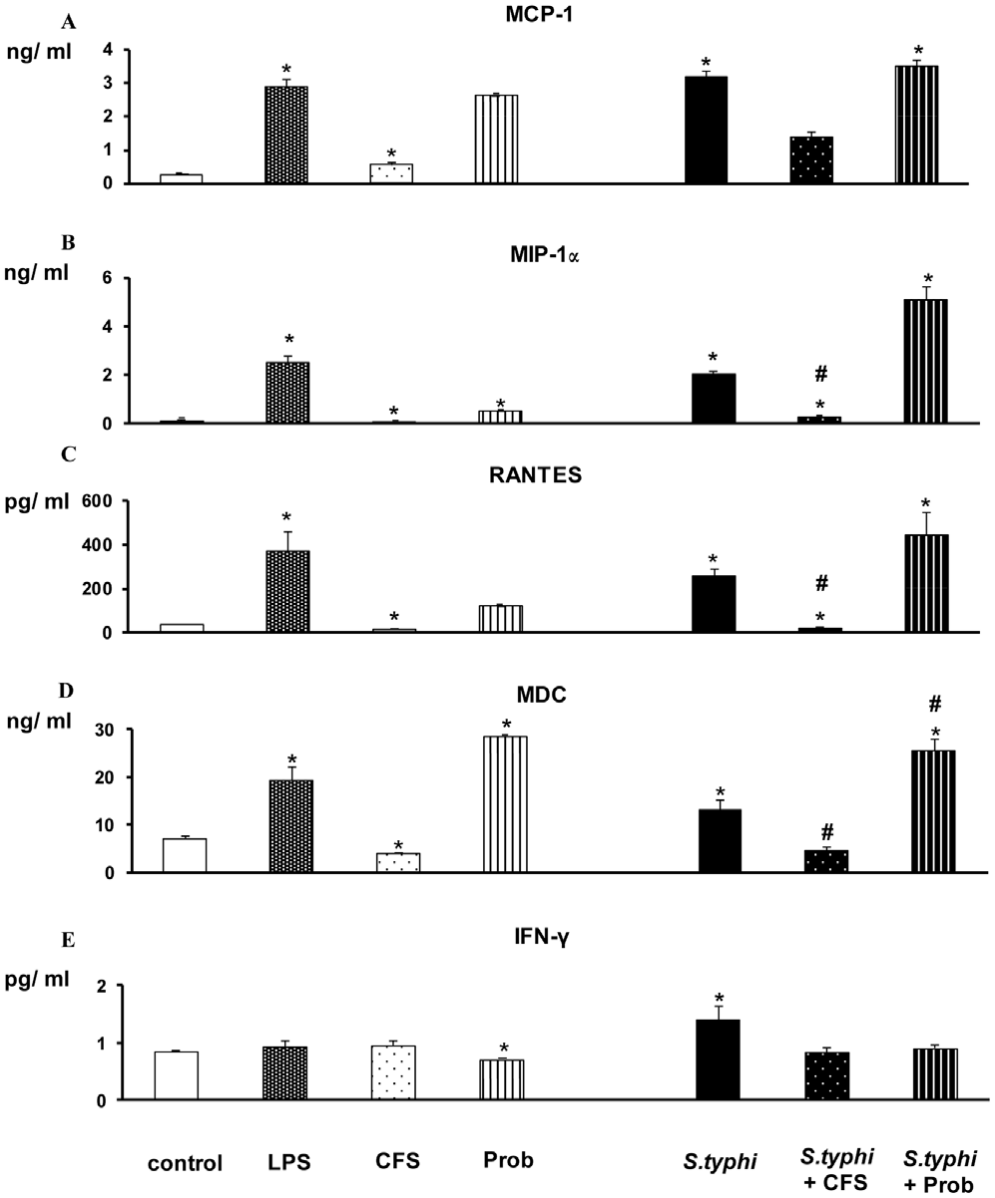
Measurement of chemokines and IFNγ in DCs after exposure to *B. breve*, *Salmonella* or a combination of the two. Dendritic cells (DCs) were incubated for 4 h with the *B. breve* CNCM I-4035 (Prob) probiotic or its cell-free supernatant (CFS), *Salmonella* (Sal) or a combination of the two and further incubated for 20 h in medium containing antibiotics. *E. coli* lipopolysaccharide (LPS; 20 ng/ml) was used as a positive control. Negative control cultures contained unstimulated DCs. Culture supernatants were collected, and the cytokine and chemokine levels were assessed by an immunoassay. The production of IFNγ and the chemokines MCP-1/CCL2, MIP-1α/CCL3, RANTES/CCL5 and MDC/CCL22 was measured. The data shown are the mean values ± SEM of three independent experiments. *, p<0.05 compared with controls; #, p<0.05 compared with *S.typhi*; N.D. indicates not detected.

The addition of pathogenic bacteria (*S. typhi* CECT 725) or LPS to DCs markedly affected the expression of pro-inflammatory cytokines (Figures 1 and 2). These treatments lead to a strong secretion of IL-1β, IL-6, IL-8, IL-12p40 and TNF-α compared to the controls. Accordingly, as illustrated in figure 3, the release of MCP-1, MIP-1α, RANTES and MDC to the culture medium was significantly elevated by either *Salmonella* or LPS stimulation.

In DCs, *B.breve* CNCM I-4035 (live bacteria) and its CFS exerted different behaviors with regard to cytokine induction in response to *B. breve* CNCM I-4035 stimulation. The CFS decreased the release of pro-inflammatory cytokines (e.g., IL-6 and IL-12p40) and chemokines (e.g., RANTES/CCL5 and MIP-1α/CCL3) in human intestinal DCs challenged with *S. typhi* (Figures 1 to 3). In contrast, the *B. breve* CNCM I-4035 strain (live bacteria) was a potent inducer of pro-inflammatory cytokines (e.g., IL-8 and IL-6; Figures 1 and 2), chemokines (e.g., MDC/CCL22; Figure 3) and some anti-inflammatory cytokines (e.g., IL-10; Figure 2). Moreover, DCs interacting with the CFS, in absence of pathogenic bacteria, released low amounts of pro-inflammatory cytokines (e.g., IL-6 and IL-12p40) and chemokines (e.g., MDC and RANTES). In contrast, *B.breve* (live bacteria) stimulation increased overall cytokine and chemokine production, namely IL-6, IL-8 and MDC (Figure 1 to 3). In addition that treatment also produced high levels of IL-10 (Figure 2).

As shown in Figure 2, the *Bifidobacterium* strain was a potent TGF-β1 inducer. Interestingly, CFS restored TGF-β levels in the presence of *Salmonella*. In contrast, live *B.breve* was unable to increase TGF-β1 production. Finally, we did not detect TGF-β2 and TGF-β3 expression.

The effects of live *B.breve* CNCM I-4035 or its CFS, *S.typhi*, or a combination of the two on the production of pro-inflammatory cytokines, anti-inflammatory cytokines and chemokines by intestinal-like human DCs are summarized in tables 1, 2 and 3, respectively. The data shown are the mean value ± SEM of three independent experiments.

**Table 1:** Effects of live *B.breve* CNCM I-4035 (Prob) or its cell-free culture supernatant (CFS), *S.typhi*, or a combination of the two on the secretion of IL-1β, IL-6, IL-8, IL-12p40 and IL-12p70 by intestinal-like human dendritic cells.

Treatment	IL-1β	IL-6	IL-8	IL-12p40	IL-12p70
Control	0,83± 0,05	1,46± 0,08	1629± 177,0	0,9± 0,36	1,49± 0,09
DCs + CFS	1,58± 0,08	10,2± 1,89	8214± 1405	0,14± 0,00	1,50± 0,12
DCs + Prob	1,81± 0,00	220,5± 0,10	16193± 125,6	3,65± 0,00	1,27± 0,05
DCs+ *S.typhi*	5,76± 1,43	396,8± 42,49	13794± 553,3	9,82± 2,54	1,69± 0,11
DCs*+* *S.typhi*+ CFS	2,49± 0,11	96,1± 10,11	13573± 2131	0,43± 0,29	1,37± 0,02
DCs*+* *S.typhi*+Prob	5,00± 0,97	526,4± 50,8	21936± 650,5	8,89± 1,23	1,74± 0,30

The data shown are the mean value ± SEM of three independent experiments.

**Table 2:** Effects of live *B.breve* CNCM I-4035 (Prob) or its cell-free culture supernatant (CFS), *S.typhi*, or a combination of the two on the secretion of IL-10, TNF-α and TGF-β1 by intestinal-like human dendritic cells.

Treatment	IL-10	TNF-α	TGF-β1
Control	1,75±0,02	9,82±1,28	319,2±48,4
DCs + CFS	1,66±0,05	42,6±3,31	252,1±50,6
DCs + Prob	14,9±0,00	85,1±1,11	216,2±77,2
DCs+*S.typhi*	15,8±2,46	244,3±47,9	157,9±11,5
*DCs+S.typhi*+CFS	4,70±0,67	264,9±57,5	273,9±33,3
*DCs+S.typhi*+Prob	46,5±7,10	377,7±80,8	143,5±25,4

The data shown are the mean value ± SEM of three independent experiments.

**Table 3:** Effects of live *B.breve* CNCM I-4035 (Prob) or its cell-free culture supernatant (CFS), *S.typhi*, or a combination of the two on the secretion of chemokines MCP-1, MIP-1α, RANTES, MDC and IFN-γ by intestinal-like human dendritic cells.

Treatment	MCP-1	MIP-1α	RANTES	MDC	IFN-γ
Control	245,5± 8,5	131,8± 15,7	37,3± 2,4	6953± 589,6	0.84± 0,02
DCs + CFS	556,4± 94,0	50,3± 1,4	13,7± 1,5	3936± 105,7	0,95± 0,08
DCs + Prob	2624± 82,2	511,8± 30,8	120,3± 2,4	28472± 89,3	0,70± 0,00
DCs+ *S.typhi*	3201± 164,4	2043,1± 86,2	258,5± 28,8	13136± 2041	1,40± 0,22
*DCs+* *S.typhi*+CFS	1372± 179,0	263,3± 29,4	19,2± 3,7	4535±8 11,0	0,83± 0,08
*DCs+* *S.typhi*+Prob	3495± 185,0	5078± 535,1	442,4± 107,3	25523± 2433	0,90± 0,07

The data shown are the mean value ± SEM of three independent experiments.

### Differences between *B. breve* CNCM I-4035 and its supernatant were observed in the induction of TLR signaling pathway components in human DCs, particularly TLR9 expression

**Figure 4:**
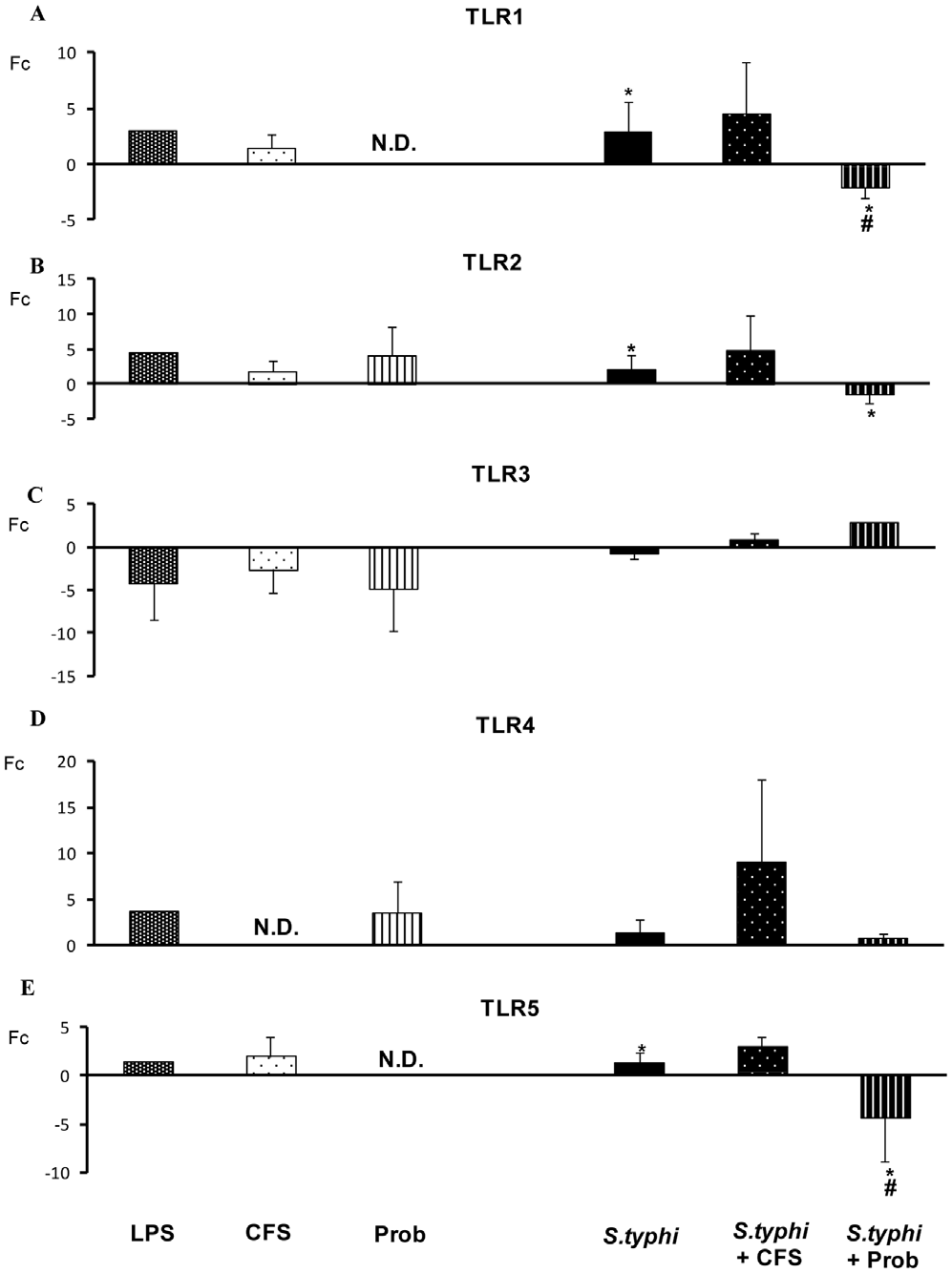
Expression of *TLR* genes in DCs in the presence of *B. breve*, *Salmonella* or a combination of the two. Comparison of the expression of *TLR1, TLR2, TLR3, TLR4* and *TLR5* in dendritic cells (DCs) in the presence of the probiotic (Prob), its supernatant (CFS), *Salmonella* (Sal) or a combination of these stimuli. *E. coli* lipopolysaccharide (LPS; 20 ng/ml) was used as a positive control. The fold change (Fc) represents the ratio of the expression in treated DCs to that in control cells. *, p<0.05 compared with controls; #, p<0.05 compared with *S. typhi.* N.D. indicates not detected.

**Figure 5:**
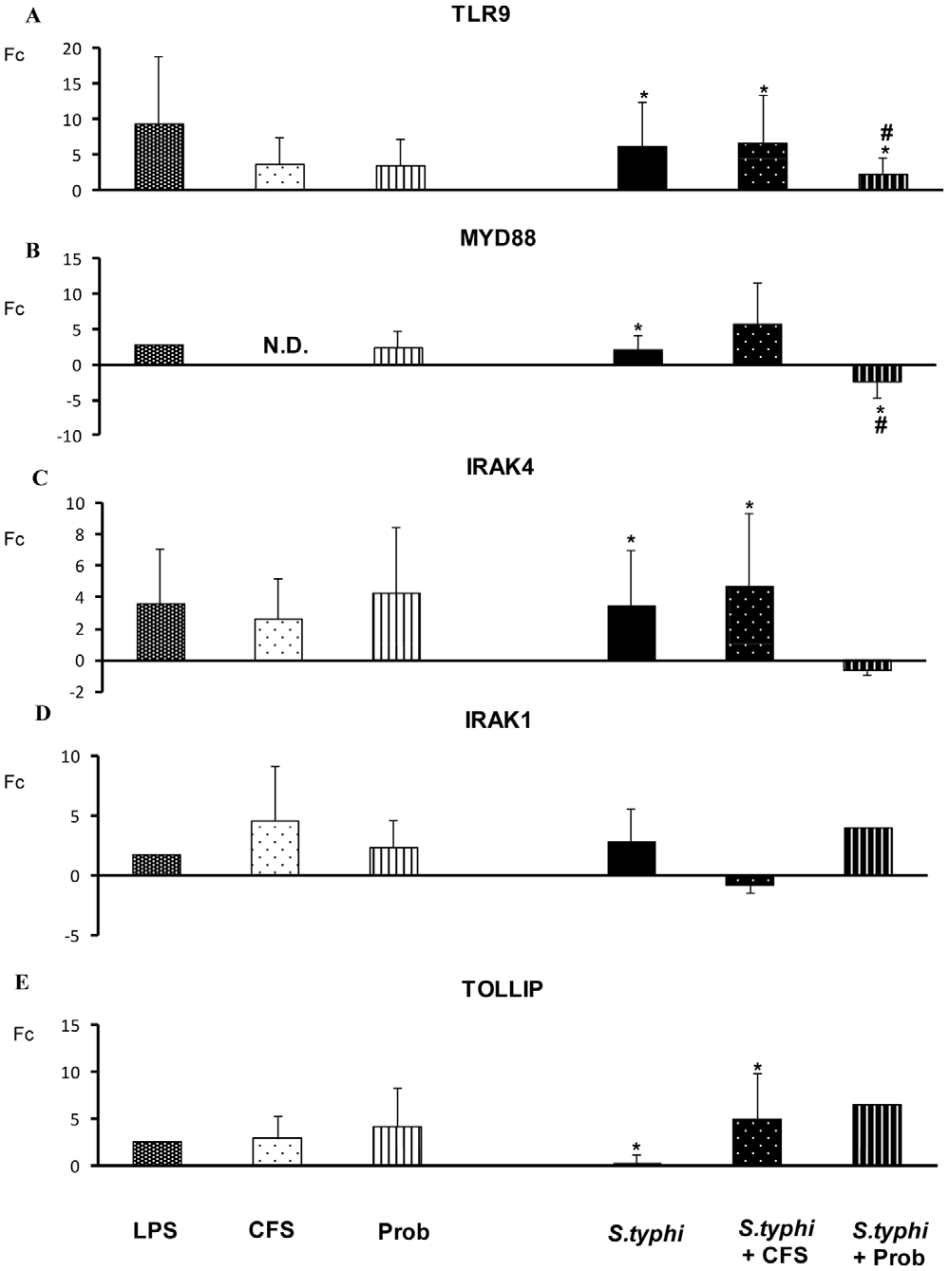
Expression of TLR signaling pathway components in DCs treated with *B. breve*, *Salmonella* or a combination of the two. Comparison of the expression of *TLR9, MYD88, IRAK-1, IRAK-4* and *TOLLIP* in dendritic cells (DCs) in the presence of the probiotic (Prob) *B. breve*, its supernatant (CFS), *Salmonella* (Sal) or a combination of these stimuli. *E. coli* lipopolysaccharide (LPS; 20 ng/ml) was used as a positive control. The fold change (Fc) represents the ratio of the expression in treated DCs to that in control cells. *, p<0.05 compared with controls; #, p<0.05 compared with *S. typhi.* N.D. indicates not detected.

**Figure 6:**
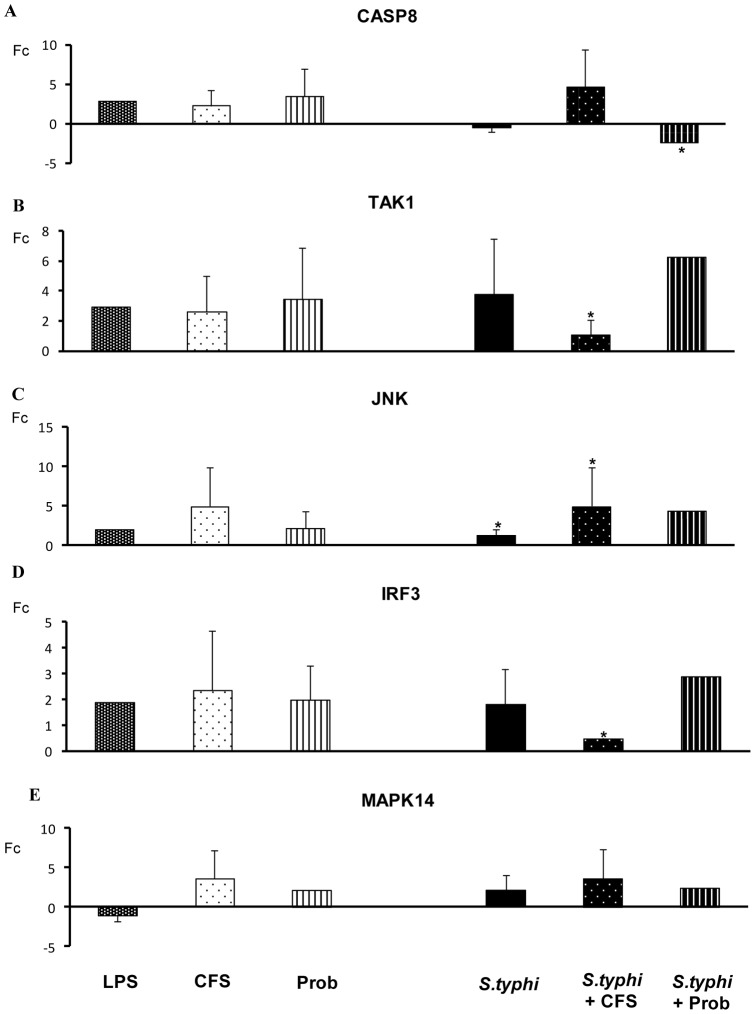
Expression of TLR signaling pathway components in DCs treated with *B. breve*, *Salmonella* or a combination of the two. Comparison of the expression of *CASP8, TAK-1, JNK, IRF-3* and *MAPK14* in dendritic cells (DCs) in the presence of the probiotic (Prob) *B. breve*, its supernatant (CFS), *Salmonella* (Sal) or a combination of these stimuli. *E. coli* lipopolysaccharide (LPS; 20 ng/ml) was used as a positive control. The fold change (Fc) represents the ratio of the expression in treated DCs to that in control cells. *, p<0.05 compared with controls; #, p<0.05 compared with *S. typhi.* N.D. indicates not detected.

**Figure 7:**
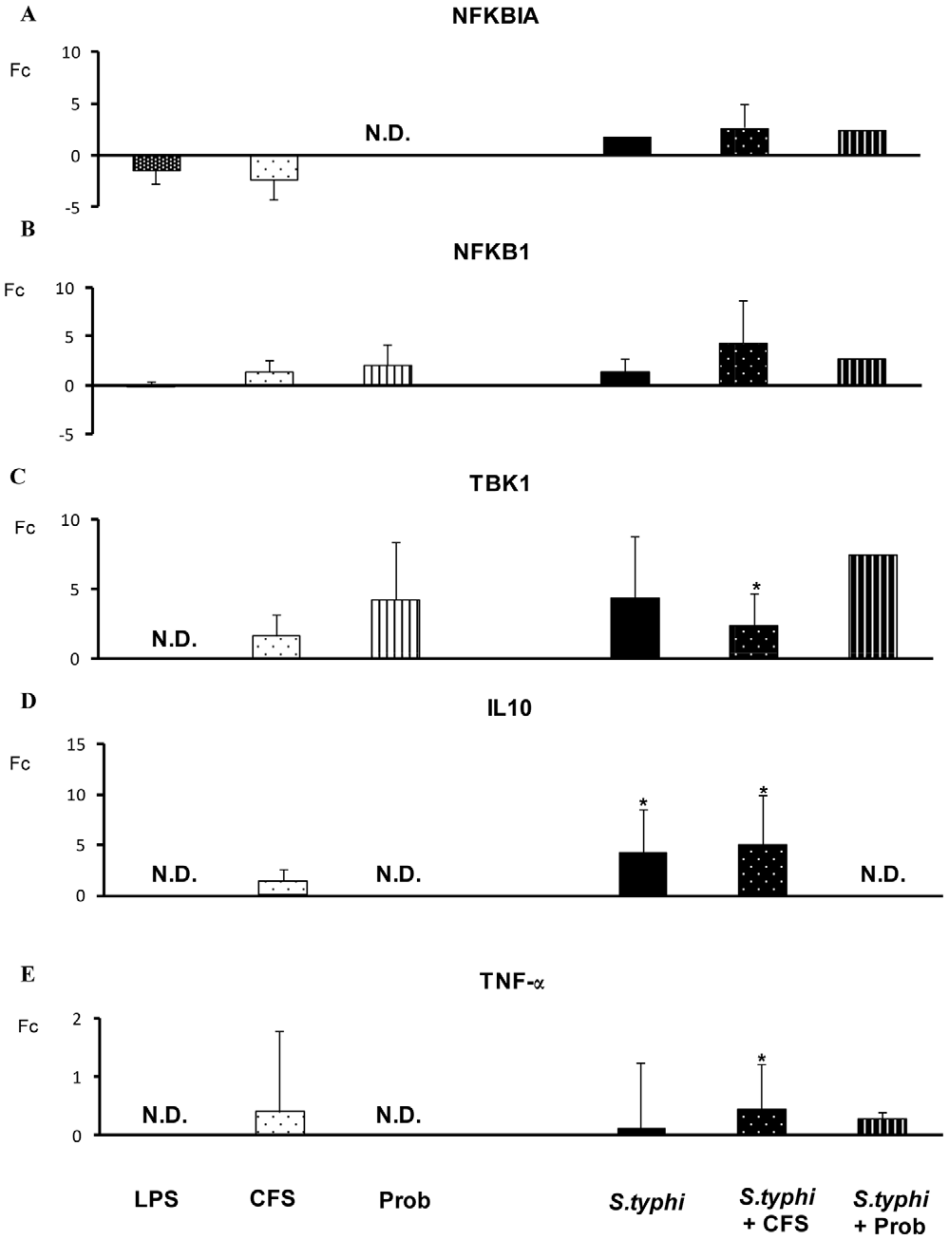
Expression of TLR signaling pathway components in DCs treated with *B. breve*, *Salmonella* or a combination of the two. Comparison of the expression of *NFKBIA, NFKB-1, TBK-1, IL-10* and *TNF-*α in dendritic cells (DCs) in the presence of the probiotic (Prob) *B. breve*, its supernatant (CFS), *Salmonella* (Sal) or a combination of these stimuli. *E. coli* lipopolysaccharide (LPS; 20 ng/ml) was used as a positive control. The fold change (Fc) represents the ratio of the expression in the treated DCs to that in the control cells. *, p<0.05 compared with controls; #, p<0.05 compared with *S. typhi.* N.D. indicates not detected.

*S. typhi* induced the expression of other TLR genes including *TLR1, TLR2* and *TLR5* (Figure 4) and upregulated *TLR9* gene expression in human DCs (Figure 5) and. A similar effect was observed for *IRAK4, TAK1, JNK* (Figures 5 and 6) and *IL-10* (Figure 7).

Differences between the probiotic bacteria *B. breve* CNCM I-4035 and its CFS were observed with regard to TLR expression in DCs (Figures 4 and 5). Both stimuli induced strong *TLR9* transcription. In addition, CFS was a more potent inducer of *TLR9* expression than live *B. breve* CNCM I-4035 in the presence of *S. typhi* (Figure 5). CFS and live *B. breve* CNCM I-4035 both induced strong and sustained *TLR2* transcription (Figure 4). The live bacteria upregulated *TLR4*, whereas CFS upregulated *TLR1* and *TLR5* (Figure 4).

Interestingly, in response to stimulation with strain *B. breve* CNCM I-4035 and *Salmonella*, *TLR1*, *TLR2* and *TLR5* expression was decreased, whereas exposure of the DCs to the probiotic and *Salmonella* upregulated *TLR3* gene expression (Figures 4 and 5). Upon stimulation with CFS plus *Salmonella*, the expression of the TLR genes increased (Figures 4 and 5). Both treatments induced the expression of *TOLLIP* (Figure 5), *JNK* (Figure 6), *TBK1* and *TNF-α* (Figure 7). We also observed differences between the treatments. CFS induced the expression of *IRAK4*, *MYD88* (Figure 5) and *CASP8* (Figure 6), whereas these genes were downregulated by *B. breve* CNCM I-4035.

## Discussion

Intestinal DCs, are known to sample microbes that continuously bombard the intestinal mucosa via PRRs such as TLRs and NLRs [25]. However, the underlying mechanisms are poorly understood. One main difficulty is the assessment of the interaction with intestinal immune system components, particularly DCs, which are key players in mucosal immunity [26]. A few studies have addressed the effects of bifidobacteria on human immunocompetent cells [27]–[29]; however, to the best of our knowledge, this is the first study to analyze the immune response to human intestinal-like DCs developed from CD34+ progenitor cells isolated from the umbilical cord blood.

The main finding of this study was that *B. breve* CNCM I-4035 and its supernatant could modify the release of cytokines by DCs in specific and differing manners. The CFS exhibited an anti-inflammatory behavior by decreasing pro-inflammatory cytokines (e.g., IL-6 and IL-12p40) and chemokines (e.g., RANTES/CCL5 and MIP-1α/CCL3) in DCs challenged with *S. typhi*. However, CFS did not increase IL-10 production. This observation is in contrast with that of Hoarau *et al.*, who reported that the *B. breve* C50 supernatant induces high IL-10 levels in DCs [30]. It is important to note that the *B. breve* supernatant by itself was a poor cytokine inducer (both inflammatory and non-inflammatory) in our study but had an important impact on the ability of human DCs to secrete lower levels of inflammatory cytokines in response to *Salmonella*, which suggests that this supernatant may have immunomodulatory properties and may be used to dampen inflammatory responses. These results are consistent with a recent study indicating similar effects for CFS from a novel probiotic strain isolated from the feces of newborns that were exclusively breast-fed (*Lactobacillus paracasei* CNCM I-4034), i.e., decreased pro-inflammatory cytokines and chemokines in human DCs challenged with *S. typhi* [23], [31]. Altogether, these data indicate that soluble bacteria product(s) released by *B.breve* CNCM I-4035 possess anti-inflammatory activity and should be identified. Work in progress in our lab using a proteomic view indicates that this bacterium secretes a number of proteins able to interact with the gut associated immune system (unpublished data).

In contrast to CFS, *B. breve* CNCM I-4035 (live bacteria) was a potent inducer of the pro-inflammatory cytokines (e.g., IL-8) and chemokines tested (e.g., MDC/CCL22). Our results are in agreement with several studies that reported that some members of the *Bifidobacterium* genus are inducers of IL-6 [32] and, to a lesser degree, IL-12 [33]. Similarly to most *Bifidobacterium* strains, live *B. breve* CNCM I-4035 stimulated the production of high levels of IL-10 [34]. This increase may have an anti-inflammatory effect [35]. The co-incubation of DCs with live *B. breve* and *S. typhi* strongly increased the release of pro-inflammatory cytokines, particularly IL-6, IL-8 and TNF-α, as well as IL-10. IL-10 and TNF-α are pleiotropic cytokines that are produced by immune cells. These cytokines are mutually regulated and play opposing roles in inflammatory responses; therefore, their relative balance is of central relevance for controlling immune deviation [36], [37]. We observed that IL-10 production was higher than TNF-α release; therefore, it appears that *B. breve* CNCM I-4035 (live bacteria) promotes anti-inflammatory effects to restore homeostasis and prevents *Salmonella*-induced inflammation. In addition, it has been suggested that the high genomic cytosine and guanosine (CG) content of the *Bifidobacterium* species (approximately 60%) increases IL-10 production [38]. Moreover, in line with other studies, live *B. breve* CNCM I-4035 did not stimulate IL-12 p70, which is a characteristic effect of bifidobacteria. However, IL-12p40 expression was increased in the presence of *S. typhi*. This result was predictable because IL-12 induction is strongly correlated with TNF-α production [32].

TGF-β expression analysis demonstrated that the *Bifidobacterium* strain is a potent TGF-β1 inducer. CFS restored TGF-β levels in the presence of *Salmonella*. TGF-β1 is a pleiotropic cytokine known to inhibit immune responses at several levels including inhibition of T cell proliferation and differentiation [39], [40] and inhibition of DC maturation [41]. Therefore, the elevation in TGF-β1 production could be responsible for the observed anti-inflammatory effects upon probiotic stimulation.

TLRs are pattern recognition receptors that recognize microbial components and initiate an innate immune response (4). *B. breve* and its supernatant possess different abilities to regulate the TLR signaling pathway. Our results demonstrate that live *B. breve* CNCM I-4035 and its CFS stimulated *TLR9* expression in the presence and absence of *Salmonella*. Plantinga *et al.* reported that cytokine induction by *B. breve* and lactobacilli is strongly dependent on TLR9 [42]. Their genomic DNA was identified as one of the anti-inflammatory components [43]. Ghadimi *et al.* reported that TLR9 signaling may at least in part mediate the anti-inflammatory effects of natural-commensal origin DNA [44]. Our results, consistent with several authors, indicate that TLR9 activation is one of the major pathways responsible for the anti-inflammatory effects of probiotics [43]–[45]. In our study, *TLR9* gene expression in response to the bacterial supernatant was significantly higher than that in response to *B. breve* CNCM I-4035 in DCs challenged with *Salmonella*. In consequence, this could explain the anti-inflammatory effects of the CFS compared with *B.breve* (live bacteria). Moreover, it has been proposed that the high frequency of CpG motifs in the DNA of the *Bifidobacterium* genus may play an important role in the immunostimulatory properties of commensal or probiotic bifidobacterial strains.

The strong upregulation of the *TLR2* gene in the presence of *B. breve* is not surprising because peptidoglycan and lipoteichoic acid, components of the cell wall of Gram-positive bacteria are TLR-2 ligands. A recent study reported that TLR2 recognition had the opposite effect of TLR9 recognition as it induced the expression of TNF-α, IL-1β and IFNγ [42]. This effect may explain the cytokine profile induced by the bacteria in the absence of *Salmonella*, which is characterized by IL-10 and TNF-α secretion. Moreover, TLR2 has also been implicated in the induction of regulatory T-cell responses, which further emphasizes the immunosuppressive potential of TLR2 signaling. However, the involvement of TLR2 in this process remains unclear, although an immunoregulatory role of TLR2 in the recognition of probiotic strains has been described [30], [46], [47].

In line with several previous studies, our results suggest that probiotic live bacteria increase the expression of *TLR2* and *TLR9* to activate an innate immune response [48]–[51] and provide immunostimulation, whereas its CFS increases the expression of *TLR9* and *TLR5* to exert anti-inflammatory effects. In addition, Uematsu *et al*. [52] proposes that microbiota induce Ig A production through a mechanism mediated by TLR5. The major role of Ig A is to maintain a balance between the host and microbiota [53], [54].

In contrast, in the presence of *Salmonella*, the CFS increased the expression of *CASP8* and *IRAK4*, whereas these genes were downregulated by the live bacteria. Both treatments induced *TOLLIP* gene expression. In this context, signal propagation is necessary to amplify the initiating signal to trigger the nuclear mobilization of transcription factors and induce gene expression. TOLLIP is an adaptor molecule that can bind to TLR2 and TLR4 to inhibit MyD88 binding and activation [55], [56]. In addition, TOLLIP binds to and is phosphorylated by IRAK, which suppresses its ability to function in the TLR pathway [57].

Finally, our results coincide with those of another study indicating that live probiotic bacteria affect the intestinal immune response, whereas secreted components exert anti-inflammatory effects in the gastrointestinal tract [58]. This supernatant may protect immune system from highly infectious agents such as *Salmonella typhi* and can down-regulate pro-inflammatory pathways.
